# Efficacy and Safety of Acupuncture in Renal Colic Caused by Urinary Calculi in Adults: A Systematic Review and Meta-Analysis

**DOI:** 10.1155/2022/7140038

**Published:** 2022-07-04

**Authors:** Zhicheng Qu, Tianyuan Wang, Jianfeng Tu, Weihai Yao, Xiaolu Pei, Liancheng Jia, Ying Cao, Cunzhi Liu

**Affiliations:** ^1^Beijing Hospital of Traditional Chinese Medicine, Capital Medical University, Beijing 100010, China; ^2^Beijing Institute of Traditional Chinese Medicine, Beijing 100010, China; ^3^International Acupuncture and Moxibustion Innovation Institute, School of Acupuncture-Moxibustion and Tuina, Beijing University of Chinese Medicine, Beijing 100029, China

## Abstract

**Background:**

Acute renal colic caused by urinary calculi has a considerable impact on the quality of life. Pain relief is the primary goal in the management of patients with acute renal colic caused by urinary calculi. At present, there is no systematic evaluation of the efficacy and safety of manual acupuncture in the treatment of acute renal colic caused by urinary calculi in adults.

**Objective:**

To evaluate the efficacy and safety of manual acupuncture in the treatment of acute renal colic caused by urinary calculi in adults.

**Methods:**

Databases of PubMed, EMBASE, Cochrane Library, China National Knowledge Infrastructure (CNKI), Wanfang Medical, VIP Database for Chinese Technical Periodicals (VIP), and China Biomedical Literature (SinoMed) were searched for literature and other randomized controlled registration platforms. We searched to identify the relevant randomized controlled trials from the establishment of the database to February 9, 2022. Only randomized controlled trials (RCTs) of manual acupuncture as the therapy for acute renal colic caused by urinary calculi in adults were included, whether or not the blind method is used. The patients were adults diagnosed with urinary calculi and renal colic. The control group was treated with commonly used analgesics and antispasmodics. The experimental group was treated with acupuncture as a monotherapy or as an adjuvant therapy (manual acupuncture combined with analgesics and antispasmodics). Two review authors independently assessed titles and abstracts for relevance and extracted data on study design, participants, interventions, and outcomes from potentially relevant articles. Cochrane risk bias assessment tool was used to evaluate the quality of the included study, and RevMan5.4 software was used for meta-analysis. Our primary outcomes were response rate and time duration before pain remission. Secondary outcomes were the time of complete pain relief, pain variation, need for rescue analgesia, and adverse events.

**Results:**

Out of 1123 records identified, 15 were found to be of relevance to this study, and 1210 participants were included in the meta-analysis. The meta-analysis of the results shows that, in terms of response rate, compared with the control group, acupuncture as a monotherapy seems to have a slight advantage (RR = 1.10 (95% CI: 1.03, 1.18), *I*^2^ = 28%, *P*=0.004), while acupuncture as an adjuvant therapy has no advantage (RR = 1.06 (95% CI: 0.95, 1.20), *I*^2^ = 77%, *P*=0.30). In terms of duration before pain relief, acupuncture as a monotherapy had an advantage over the control group (MD = −10.28(95% CI: −14.40, −6.17), *I*^2^ = 93%, *P* < 0.00001). Acupuncture as a monotherapy was similar to positive medication in terms of complete pain relief (MD = −7.13 (95% CI: −20.19, 5.94), *I*^2^ = 95%, *P*=0.28). Pain variation: VAS scores at 10 min, acupuncture as a monotherapy (MD = −2.47 (95% CI: −3.40, −1.53), *I*^2^ = 84%, *P* < 0.00001) or as an adjuvant therapy (MD = −3.38 (95% CI: −4.33, −2.43), *I*^2^ = 60%, *P* < 0.00001) was better than the control group. VAS scores at 30 min, compared with the control group, there was no difference between acupuncture as a monotherapy (MD = −0.27 (95% CI: −1.43, 0.88), *I*^2^ = 88%, *P*=0.64) and acupuncture as an adjuvant therapy (MD = −1.17 (95% CI: −3.15, 0.81), *I*^2^ = 96%, *P*=0.25). VAS scores at 60 min, compared with the control group, there was no difference in the acupuncture as a monotherapy (MD = 0.58 (95% CI: −0.28, 1.45), *I*^2^ = 77%, *P*=0.19), while acupuncture as an adjuvant therapy was better (MD = −1.22 (95% CI: −1.93, −0.51), *I*^2^ = 72%, *P*=0.0007). VAS scores at 120 min, there was no difference in acupuncture as a monotherapy compared to the control group (MD = −0.24 (95% CI:−1.22, 0.75), *I*^2^ = 0, *P*=0.64). One study reported on rescue analgesia. Fewer adverse events occurred in the experimental group compared to the control group.

**Conclusion:**

In the course of manual acupuncture treatment of acute renal colic caused by urinary calculi in adults, available evidence suggests that manual acupuncture is as effective as positive treatment drugs, either as a monotherapy or as an adjunctive therapy, with the advantage of acupuncture being its rapid onset of action. However, the number of existing clinical studies is small, and the quality of evidence is generally low, so it is recommended to use it with caution. In order to further verify the above conclusions, more high-quality clinical RCTs need to be carried out. *Trial Registration*. The present review protocol was registered with the International Prospective Register of Systematic Reviews (CRD42019134900).

## 1. Introduction

Renal colic is a common and painful condition in emergency departments, with millions of patients presenting as a result each year worldwide. The incidence of urinary stones varies widely worldwide, ranging from 1% to 20% due to factors such as geography, climate, ethnicity, diet, fluid intake, genetics, gender, occupation, and age [1]. The National Health Service, England statistics for the year 2012–2013 estimated the cost for renal colic at nearly £20 million, where the median patient stay in the hospital was 1 day [2].

As patients with renal colic are extremely distressed, one of the first priorities in the management of renal colic is to provide quick, safe, and effective analgesia. However, quick and effective analgesia can be practically challenging to deliver in emergency departments with a diverse population and a high volume of patients being managed concurrently [3]. According to the research of the European Association of Urology (EAU), nonsteroidal anti-inflammatory drugs and opioids are commonly used as analgesics in clinics [4]. The application of traditional Chinese medicine acupuncture has a history of thousands of years, and it has been widely used in the treatment of pain diseases. There is substantial evidence that acupuncture is effective in the treatment of acute and chronic pain [5–7]. It is mainly achieved in two ways: one is to correct and eliminate the pathological factors that produce pain, and the other is to block the bad circulation of pain. The two complement each other and play a common role [8]. However, in recent years, there have also been some skeptical voices that acupuncture is ineffective or not superior to placebo [9–11]. Studies in recent years have shown that the acupuncture treatment of acute renal colic caused by urinary calculi has rapid pain relief [12, 13], reliable curative effect, and no toxic side effects, but the sample sizes of these trials were small, and their conclusions were seen as overly positive. At present, over 80 systematic reviews have been conducted to assess the role of acupuncture and related therapies in the relief of pain; however, the results of these systematic reviews are far from unanimous [14], and there is no systematic evaluation of the efficacy and safety of manual acupuncture in the treatment of acute renal colic caused by urinary calculi in adults. The purpose of this study is to systematically evaluate the efficacy and safety of manual acupuncture in the treatment of acute renal colic caused by urinary calculi in adults and to provide more evidence for clinical application.

## 2. Methods

The present systematic review and meta-analysis conforms to the Preferred Reporting Items for Systematic reviews and Meta-Analyses reporting guideline [15]. The present review protocol was registered with the International Prospective Register of Systematic Reviews (PROSPERO registration number ID 42019134900). The protocol was not published.

### 2.1. Retrieval Strategy

We searched the database of Cochrane Library, EMBASE, PubMed, CNKI, Wanfang Medical Database, SinoMed, and VIP from the establishment of the database to February 9, 2022 and collected randomized controlled trials on manual acupuncture treatment of acute renal colic caused by urinary calculi in adults. We also searched the WHO International Clinical Trials Registry Platform, ClinicalTrials.gov, and Chinese Clinical Trial Registry. According to the characteristics of their respective databases, two researchers (ZC Qu and Y Cao) adopted free words and subject words and used the following keywords to conduct a comprehensive search of the full text, topics, and abstract. The search strategies include (Renal Colic OR Colic, Renal OR Colics Renal OR Renal Colics OR Acute Renal Colic OR Acute Renal Colics OR Colic, Acute Renal OR Colics, Acute Renal OR Renal Colic, Acute OR Renal Colics, Acute OR Ureteral Colic OR Colic, Ureteral OR Colics, Ureteral OR Ureteral Colics) AND (Acupuncture OR Pharmacopuncture OR Acupuncture Therapy OR Therapy, Acupuncture OR Acupuncture Treatment OR Acupuncture Treatments OR Treatment, Acupuncture OR Pharmacoacupuncture Treatment OR Treatment, Pharmacoacupuncture OR Pharmacoacupuncture Therapy OR Therapy, Pharmacoacupuncture OR Acupuncture Points OR Acupuncture Point OR Point, Acupuncture OR Points, Acupuncture OR Acupoints OR Acupoint OR Acupuncture Analgesia OR Analgesia, Acupuncture OR Acupuncture Anesthesia OR Anesthesia, Acupuncture).

### 2.2. Inclusion Criteria

Only RCTs of manual acupuncture as the therapy for acute renal colic caused by urinary calculi in adults were included, whether or not the blind method is used. The patients were adults diagnosed with renal colic caused by urolithiasis. The control group was treated with commonly used analgesics and antispasmodics (e.g., diclofenac, indomethacin, ibuprofen, morphine, pethidine, tramadol, atropine, anisodamine, and acetaminophen). The experimental group was treated with acupuncture as a monotherapy or as an adjuvant therapy (manual acupuncture combined with analgesics and antispasmodics). The frequency, duration, language, and state of publication of treatment are not limited.

### 2.3. Exclusion Criteria

1. The study that did not specify diagnostic criteria for renal colic
2. The patient with acute pain or long-term chronic pain not caused by renal colic
3. The experimental group that was treated with auricular point acupuncture, scalp acupuncture, fire acupuncture, warm acupuncture, electroacupuncture, acupoint injection, and laser acupuncture
4. Animal research, cell experiments, reviews, retrospective studies
5. Data that are incomplete or cannot be extracted and the data that could not be obtained after contacting the author

### 2.4. Data Extraction

The retrieved literatures were screened independently by two researchers (ZC Qu and Y Cao), according to the inclusion and exclusion criteria specified in this study. Then, the document management software Endnote and office software Excel were used to create data extraction tables to manage and extract the research data, respectively, followed by cross-checking. If the same study was reported many times by different literatures, it would be included in the one with the most detailed content. When there was a difference in opinion, the two sides discussed and resolved it with the third person (TY Wang) if it could not be unified. The name of the first author, the published time, the sample size, the age of the subjects, the intervention measures, and the outcome index were mainly included. The original literature was checked or discussed if there were inconsistencies in data extraction. If necessary, the author of the study was contacted until the issue is resolved.

### 2.5. Outcomes

#### 2.5.1. Primary Outcomes

The primary outcomes were response rate and time duration before pain remission. The response rate was the number of cured cases, the number of markedly effective cases, and the percentage of the sum of effective cases to the total number of cases. VAS score scale was used to record the pain score during acupuncture. For pain variance, based on the previous literature and expert opinions [16], we pooled data from studies reporting VAS 100 mm and VAS 10 cm by converting it to a “0–10-pain measure.” VAS = 0 indicated no pain, 0 < VAS <3 indicated mild pain; 3≤ VAS <7 indicated moderate pain; and 7≤ VAS <10 indicated severe pain. The pain relief rate (PRR) is calculated using the formula: PRR = (VAS0 min-VASend)/VAS0 min) × 100%. According to PRR, the curative effects were divided into: (1) cure: the pain disappeared completely at the end of treatment, PRR = 100%; (2) markedly effective: the pain was significantly relieved at the end of treatment, 75%≤ PRR <100%; (3) effective: the pain was relieved at the end of treatment, 50%≤ PRR <75%; (4) ineffective: there was no significant improvement in pain at the end of treatment, PRR <50%.

Time duration before pain remission means the time when pain begins to relieve or the onset of pain relief.

#### 2.5.2. Secondary Outcomes

The secondary outcomes were the time of complete pain relief, pain variation (such as VAS score at 10 min, 30 min, 60 min, and 120 min), need for rescue analgesia, and adverse events.

### 2.6. Risk of Bias Assessment

Based on the Cochrane bias risk assessment tool, the risk of bias is assessed from seven aspects: random sequence generation, allocation concealment, blinding of investigators and subjects, blinded evaluation of research results, integrity of result data, selective reporting of research results, and other biases. The investigators' judgments were classified as low risk of bias, high risk of bias, and unclear risk. If the relevant details were not adequately reported in the study, the adjudication was usually “uncertain” for the risk of bias. Also, an “uncertain” judgment is made if a study reports relevant details but its risk of bias is unknown.

Blinding of personnel was so difficult, as acupuncture needs to be performed by a qualified professional. The results due to unblinding resulted in the existence of a high-risk bias; however, this should be interpreted with the knowledge that blinding is difficult in acupuncture studies.

### 2.7. Summary of Findings and Assessment of the Certainty of the Evidence

Two review authors (ZC Qu and Y Cao) independently rated the certainty of the evidence for each outcome using the GRADE system and GRADEprofiler Guideline Development Tool software (GRADEpro GDT) and the guidelines provided in Chapter 14 of the Cochrane Handbook for Systematic Reviews of Interventions. We used the five GRADE considerations (study limitations, consistency of effect, imprecision, indirectness, and publication bias) to assess the certainty of the body of evidence as it related to the studies that contributed data to the meta-analyses for the prespecified outcomes. We assessed the certainty of evidence as high, moderate, low, or very low. We considered the following criteria for upgrading the certainty of evidence, if appropriate: large effect, dose-response gradient, and plausible confounding effect. We justified all decisions to down or upgrade the certainty of studies using footnotes to aid the reader's understanding of the results where necessary.

### 2.8. Data Analysis

The meta-analysis of all data was carried out by using RevMan5.4 [Version 5.4. Copenhagen: The Nordic Cochrane Centre, The Cochrane Collaboration, 2020]. The relative risk (RR) degree was used as the effect analysis statistic for the two classification variables; the mean difference (MD) was used when the continuous variable measurement method or unit was consistent, and the standardized mean difference (SMD) was used as the effect index when it was inconsistent. Both of them calculated a 95% confidence interval (CI). The heterogeneity among the results of each study was evaluated by a Q-value test combined with *I*^2^. Heterogeneity was examined using the *I*^2^ test. Thresholds for the interpretation of the *I*^2^ statistic can be misleading, since the importance of inconsistency depends on several factors. We considered heterogeneity by a rough guide to interpretation, which is as follows [17]:

1. The *I*^2^ value: 0%–40%: represents might not be important
2. The *I*^2^ value: 30%–60%: represents moderate heterogeneity
3. The *I*^2^ value: 50%–90%: represents significant heterogeneity
4. The *I*^2^ value: 75%–100%: represents considerable heterogeneity

Sensitivity analysis was carried out to find whether the results were stable, and subgroup analysis was used to analyze the factors that may lead to heterogeneity. If the heterogeneity is too obvious, especially when clinical heterogeneity is obvious as well as the data could not be combined, descriptive analysis was used and the results were explained. We also assessed the publication bias by using a funnel plot according to the type of acupuncture.

## 3. Results

### 3.1. Search and Screening

**Figure 1:**
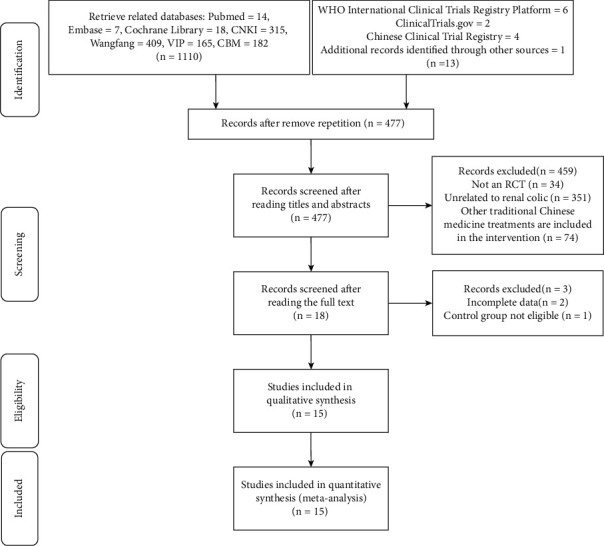
Literature screening flow diagram.

According to the retrieval strategy, 1123 related articles were searched. After removing the repetition, there are 477 articles left, 18 articles are left after reading titles and abstracts, and 15 articles are selected after reading the full text (Figure 1).

### 3.2. Study Characteristics

A total of 15 RCT studies [12, 13, 18–30] were included in this study, including 1210 subjects aged from 18 to 81—579 patients in the experimental group and 631 patients in the control group (Tables 1 and 2). There are 11 Chinese literatures and 4 English literatures. 13 studies [18–30] were carried out in China, 1 study [13] in Tunisia, and 1 study [12] in Turkey. 14 studies were published and 1 study [30] was unpublished. We contacted the authors, obtained their consent, and obtained study data. In the experimental group, 9 studies [12, 13, 19–24, 26] were treated with manual acupuncture as a monotherapy, 1 study [16] was treated with manual acupuncture combined with sham drug; 5 studies [25, 27–30] were treated with manual acupuncture as an adjunctive therapy. In the control group, 2 studies used sham acupuncture [29, 30]. All studies used analgesic and/or antispasmodics, some trials were combined with two or three kinds of drugs. 9 trials [12, 19–23, 25, 27, 29] had used opioid, 4 trials [13, 18, 26, 30] had used NSAIDs, 7 trials [19, 21, 22, 24, 27–29] had used antispasmodics, 1 trial [13] had used acetaminophen, and 1 trial [19] had used phenergan. Response rates were reported for outcome measures in 13 studies [13, 18–25, 27–30]. 5 studies [20, 22–24, 26] reported time duration before pain remission, and 2 studies [13, 20] reported time of complete pain relief. 6 studies [12, 13, 18, 23, 24, 30] reported adverse events, 1 study [23] of which did not mention specific symptoms. Details of needling are given in Table 3.

### 3.3. Quality Assessment

#### 3.3.1. Risk of Bias in Included Studies

**Figure 2:**
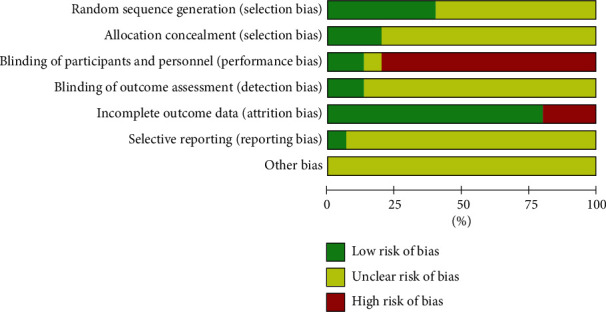
Risk of bias graph.

**Figure 3:**
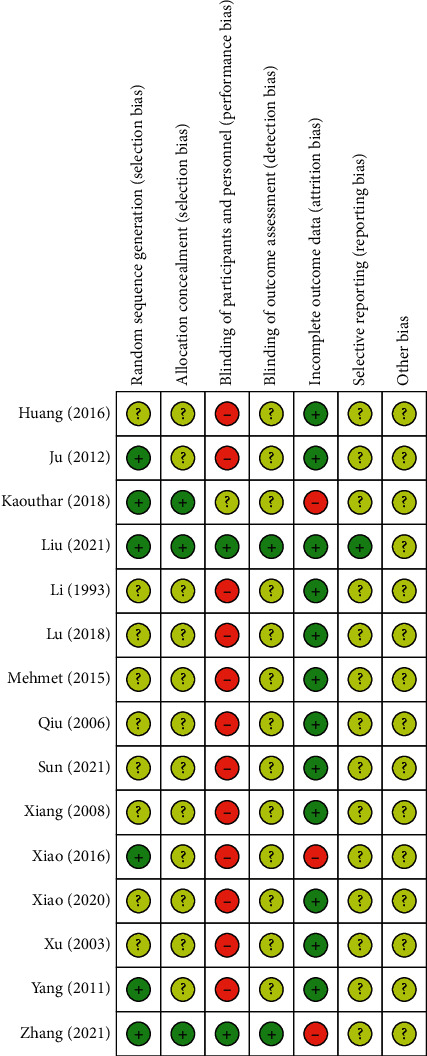
Risk of bias summary.

The risks of bias are shown in Figures 2 and 3. Random is mentioned in all studies. 6 studies [13, 18, 23, 24, 26, 30] explain the random method, which is the random number table method, the rest of the studies did not describe enough information. With the exception of 3 studies [13, 18, 30], rest of the 12 studies [12, 19–29] did not have enough description to support clear judgments on allocation concealment. Regarding the blinding of participants and personnel, with the exception of 2 studies [18, 30], the rest of included studies were judged as having a high risk of bias because they did not use sham acupuncture as the control. The blinding of outcome assessors was described in only 2 studies [18, 30] at low risk of bias; the risk of bias in others was unclear, as there was no explanation we could assess the risk of bias. With the exception of 3 studies [13, 18, 26], rest of the 12 studies [12, 19–25, 27–30] were rated low risk for complete outcome data. The risk of bias in selective reporting was assessed unclear, as study protocols were not always acquired. Other biases were assessed unclear in all studies, because we could not assess the risk of bias without any explanation.

#### 3.3.2. Assess Certainty (or Confidence) in the Body of Evidence for an Outcome

**Figure 4:**
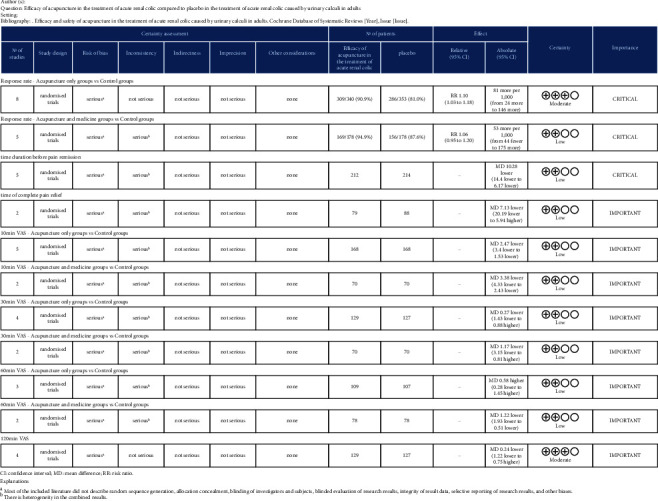
Summary of findings.

Compared with the control group, moderate-certainty evidence indicates acupuncture as a monotherapy group lead to improvements in response rate. The evidence was downgraded in one step for bias. Low-certainty evidence indicates that there was no statistical difference between the response rate of relieving renal colic by acupuncture as an adjuvant therapy group and the control group. The evidence was downgraded in two steps: once for bias and once for inconsistency caused by high heterogeneity. Compared with the control group, low-certainty evidence indicates manual acupuncture as a monotherapy group lead to reduce the time duration before pain remission. The evidence was downgraded in two steps: once for bias and once for inconsistency caused by high heterogeneity. Low-certainty evidence indicates that there was no statistically significant difference between acupuncture as a monotherapy and the control group for the time of complete pain relief. The evidence was downgraded in two steps: once for bias and once for inconsistency caused by high heterogeneity. Low-certainty evidence indicates that the VAS score of acupuncture as a monotherapy group relieving renal colic 10 min was better than the VAS score of the control group. The evidence was downgraded in two steps: once for bias and once for inconsistency caused by high heterogeneity. Low-certainty evidence indicates that the VAS score of acupuncture as an adjuvant therapy group relieving renal colic 10 min was better than the VAS score of the control group. The evidence was downgraded in two steps: once for bias and once for inconsistency caused by high heterogeneity. Low-certainty evidence indicates that there was no statistically significant difference between acupuncture as a monotherapy and the control group for VAS score at 30 min. The evidence was downgraded in two steps: once for bias and once for inconsistency caused by high heterogeneity. Low-certainty evidence indicates that there was no statistically significant difference between acupuncture as an adjuvant therapy and the control group for VAS score at 30 min. The evidence was downgraded in two steps: once for bias and once for inconsistency caused by high heterogeneity. Low-certainty evidence indicates that there was no statistically significant difference between acupuncture as a monotherapy and the control group for VAS score at 60 min. The evidence was downgraded in two steps: once for bias and once for inconsistency caused by high heterogeneity. Low-certainty evidence indicates that the VAS score of acupuncture as an adjuvant therapy group relieving renal colic 60 min was better than the VAS score of the control group. The evidence was downgraded in two steps: once for bias and once for inconsistency caused by high heterogeneity. Moderate-certainty evidence indicates that there was no statistically significant difference between acupuncture as a monotherapy and the control group for VAS score at 120 min. The evidence was downgraded in one step for bias. A summary of findings' table presents the same information as the text above, with footnotes explaining judgments (Figure 4).

### 3.4. Data Analysis

#### 3.4.1. Response Rate

**Figure 5:**
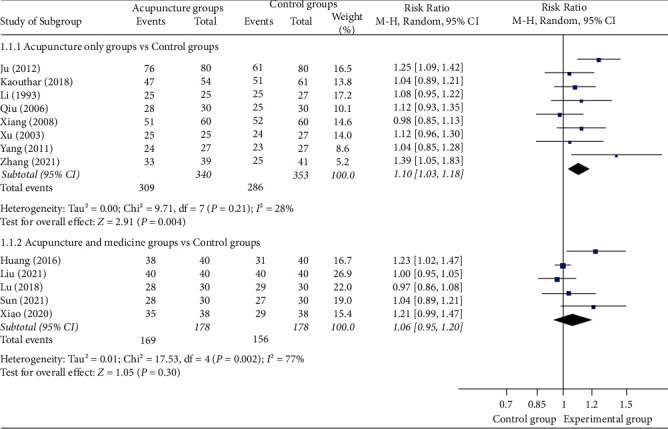
Forest plots of response rate.

A total of 13 studies [13, 18–25, 27–30] reported effective rates, of which 8 studies used manual acupuncture as a monotherapy in the experimental group and 5 used acupuncture as an adjuvant therapy in the experimental group. Meta-analysis shows that compared to the control group, 8 studies [13, 18–24] which were manual acupuncture as a monotherapy is more likely to lead to improvements in response rate (RR = 1.10(95% CI: 1.03, 1.18), *I*^2^ = 28%, *P*=0.004) with a high degree of homogeneity. Compared with the control group, acupuncture as an adjuvant therapy included 5 studies [25, 27–30], there was no statistical difference (RR = 1.06 (95% CI: 0.95, 1.20), *I*^2^ = 77%, *P*=0.30) (Figure 5).

#### 3.4.2. Time Duration before Pain Remission

**Figure 6:**
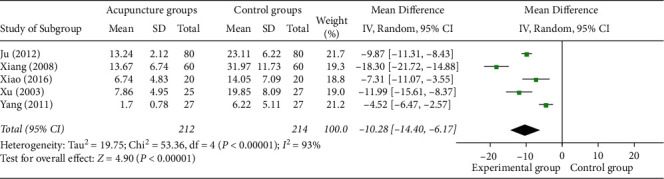
Forest plots of time duration before pain remission.

5 studies [20, 22–24, 26] reported the time duration before pain remission, all of which used manual acupuncture as a monotherapy in the experimental group. Meta-analysis shows that acupuncture as a monotherapy in renal colic is more likely to lead to reduce the time duration before pain remission (MD = −10.28 (95% CI: −14.40, −6.17), *I*^2^ = 93%, *P* < 0.00001) with severe heterogeneity, compared to the control group (Figure 6).

#### 3.4.3. Time of Complete Pain Relief

**Figure 7:**
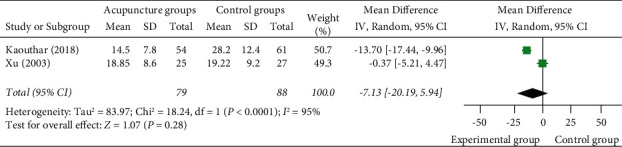
Forest plots of time of complete pain relief.

2 studies [13, 20] reported the time of complete pain relief, all of which used manual acupuncture as a monotherapy in the experimental group. Meta-analysis shows that there was no statistically significant difference between acupuncture as a monotherapy and the control group (MD = −7.13 (95% CI: −20.19, 5.94), *I*^2^ = 95%, *P*=0.28) (Figure 7).

#### 3.4.4. Pain Variation

**Figure 8:**
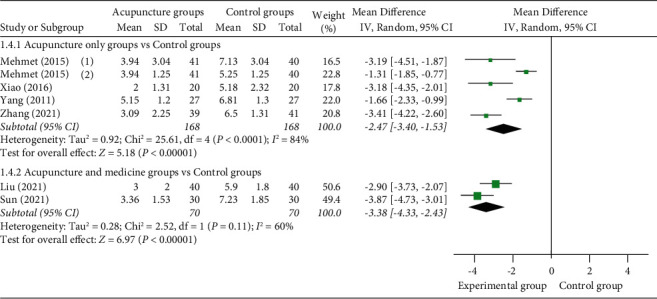
Forest plots of VAS score at 10 min.

**Figure 9:**
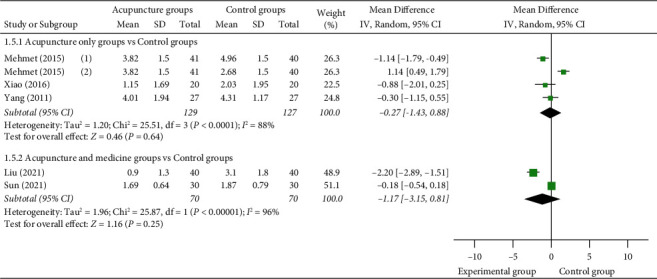
Forest plots of VAS score at 30 min.

**Figure 10:**
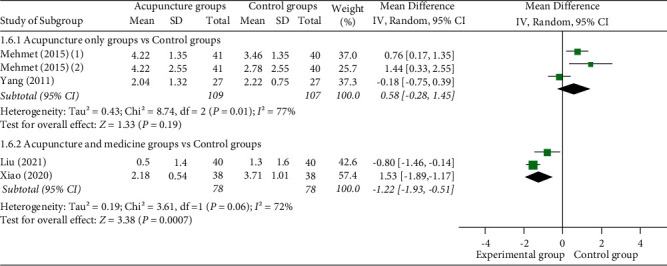
Forest plots of VAS score at 60 min.

**Figure 11:**
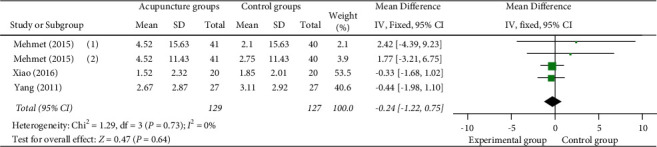
Forest plots of VAS score at 120 min.

(1) VAS score at 10 min: 6 studies [12, 18, 23, 26, 29, 30] reported VAS scores at 10 min, of which 4 studies [12, 18, 23, 26] used acupuncture as a monotherapy in the experimental group and 2 studies [29, 30] used acupuncture as an adjuvant therapy in the experimental group. 1 study [12] set up two control groups, so they were compared separately. Meta-analysis shows that the VAS score of manual acupuncture as a monotherapy group was better than the VAS score of the control group (MD = −2.47 (95% CI: −3.40, −1.53), *I*^2^ = 84%, *P* < 0.00001). The VAS score of acupuncture as an adjuvant therapy group was also better than the VAS score of the control group relieving renal colic (MD = −3.38 (95% CI: −4.33, −2.43), *I*^2^ = 60%, *P* < 0.00001) (Figure 8).

(2) VAS score at 30 min: 5 studies [12, 23, 26, 29, 30] reported VAS scores at 30 min, of which 3 studies [12, 23, 26] used acupuncture as a monotherapy in the experimental group and 2 studies [29, 30] used acupuncture as an adjuvant therapy in the experimental group. 1 study [12] set up two control groups, so they were compared separately. Meta-analysis shows that there was no statistically significant difference between manual acupuncture as a monotherapy group and the control group (MD = −0.27 (95% CI: −1.43, 0.88), *I*^2^ = 88%, *P*=0.64). There was no statistically significant difference between acupuncture as an adjuvant therapy group and the control group (MD = −1.17 (95% CI: −3.15, 0.81), *I*^2^ = 96%, *P*=0.25) (Figure 9).

(3) VAS score at 60 min: 4 studies [12, 23, 26, 30] reported VAS scores at 60 min, of which 2 studies [12, 23] used manual acupuncture as a monotherapy in the experimental group and 2 studies [29, 30] used acupuncture as an adjuvant therapy in the experimental group. 1 study [12] set up two control groups, so they were compared separately. Meta-analysis shows that there was no statistically significant difference between manual acupuncture as a monotherapy group and the control group (MD = 0.58 (95% CI: −0.28, 1.45), *I*^2^ = 77%, *P*=0.19). The VAS score of acupuncture as an adjuvant therapy group was better than the control group at 60 min (MD = −1.22 (95% CI: −1.93, −0.51), *I*^2^ = 72%, *P*=0.0007) (Figure 10).

(4) VAS score at 120 min: 3 studies [12, 23, 26] reported VAS scores at 120 min, all of which used manual acupuncture as a monotherapy in the experimental group. 1 study [16] set up two control groups, so they were compared separately. Meta-analysis shows that there was no statistically significant difference between manual acupuncture as a monotherapy group and the control group (MD = −0.24 (95% CI:−1.22, 0.75), *I*^2^ = 0, *P*=0.64) (Figure 11).

#### 3.4.5. Need for Rescue Analgesia

With the exception of 1 study [30], the rest of included studies did not address or report on rescue analgesia.

#### 3.4.6. Incidence of Adverse Reactions

6 studies [12, 13, 18, 23, 24, 30] reported adverse events, 1 study [23] of which did not mention specific symptoms. The experimental group included: 1 case of frequent urination, 1 case of needle blockage, and 2 cases of itching/rash/bleeding at insertion point. The control group included: 60 cases of dizziness, 33 cases of nausea and vomiting, 1 case of lethargy, 2 cases of rash, 13 cases of hypotension, 1 case of allergic reaction, 2 cases of abdominal burning, and 29 cases of fatigue. Compared with the experimental group, more adverse events occurred in the control group.

#### 3.4.7. Subgroup Analysis and Heterogeneity Exploration

Subgroup analysis was attempted based on these two conditions (acupuncture as a monotherapy or an adjuvant therapy). However, after discussion, we think whether the intervention of interest used as a monotherapy or as an adjuvant therapy has completely different meanings in the interpretation of the results. Therefore, we changed the plan to analyze acupuncture as a monotherapy and acupuncture as an adjuvant therapy separately for each outcome measure instead of subgroup analysis. Due to the small number of studies included in each outcome (<10), we did not set up a subgroup for analysis.

Most of the literature did not explain the basic characteristics of the included population, so it is difficult for us to judge whether the basic characteristics of the included population are consistent based on the existing data; each included literature has different treatment regimens, including acupuncture points, acupuncture depth, acupuncture technique, and needle retention time, as well as the type, dosage, and usage of the positive control drug. These may have contributed to the excessive heterogeneity in the outcome measures.

#### 3.4.8. Sensitivity Analysis

Sensitivity analysis could not be performed as planned due to the small number of literatures included in the outcome measures after separate comparisons of acupuncture as a monotherapy or an adjuvant therapy.

#### 3.4.9. Publication Bias

**Figure 12:**
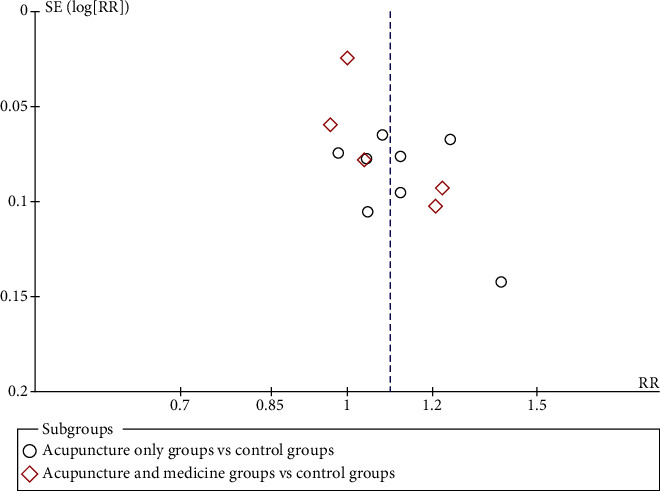
Funnel plot of response rate.

Meta-analysis of included 15 studies compared the experimental group with the control group. There was high asymmetry means there is a large publication bias according to the funnel plot (Figure 12).

## 4. Discussion

### 4.1. Summary of the Evidence

We aimed to evaluate the efficacy and safety of manual acupuncture in the treatment of acute renal colic caused by urinary calculi in adults. Based on the current level of evidence, in the treatment of renal colic, we believe that there is insufficient evidence to support that acupuncture as a monotherapy or acupuncture as an adjuvant therapy is superior in terms of overall efficacy compared with drug alone. The onset time and 10 min VAS score were better than those of the control group, suggesting that acupuncture as a monotherapy or acupuncture as an adjuvant therapy appears to have an advantage in terms of rapid onset of action (immediate pain relief), but with the passage of time, the drug gradually began to exert its curative effect, and the advantage of acupuncture gradually weakened, and this advantage almost disappeared after 30 minutes of treatment.

### 4.2. Limitations

Our study has some limitations. Outcome level: the meta-analysis reported here combines data across studies in order to estimate treatment effects with more precision than is possible in a single study. The main limitation of this meta-analysis, as with any overview, is that the patient population, acupuncture as a monotherapy or acupuncture as an adjuvant therapy regimen, and the outcome definitions are not the same across studies. Study and review level: most of the included studies were Chinese with a relatively small sample size. The quality of research was uneven. Only 3 trials reported comparatively complete details of needling. In some studies, the key information for judging the risk of bias is often not clearly explained. For example, most articles do not explicitly state that data analysis followed the intention-to-treat principle, which may lead to the overestimation of treatment effects in these trials. The evidence for the results was low. Some literatures are too old, and the selected primary outcome indicators are too subjective and lack objectivity. The included literature comparison methods are not uniform enough, which may be one of the reasons for the heterogeneity.

### 4.3. Implications for Research

The 15 included studies were carried out in 3 countries: China (Asia), Tunisia (Africa), and Turkey (Asia-Europe). The included randomized controlled trials were mainly conducted in China and reported short-term or medium-term results. As a result, there is a lack of international regional coordination mechanisms and long-term results. It is suggested to carry out large-sample and high-quality RCTs in the future, to unify the course of acupuncture treatment and acupuncture points, to avoid clinical heterogeneity as far as possible, and to select internationally recognized outcome indicators and specify unified evaluation criteria in clinical trials. The cycle of existing research is relatively short, so it is recommended to evaluate the long-term effect and the prevention of recurrence of renal colic.

## 5. Conclusion

For the treatment of adult renal colic, available evidence suggests that manual acupuncture is as effective as positive treatment drugs, either as a monotherapy or as an adjunctive therapy, with the advantage of acupuncture being its rapid onset of action. However, the overall quality of the existing research is low, and the quantity is relatively small. Many of the included trials did not fully meet the STRICTA guidelines, particularly in their failure to report the details of needling. Therefore, we recommend caution in clinical use. We suggest that future researchers carefully consult the STRICTA checklist when designing and reporting acupuncture treatment protocols. This will enable the acupuncture practices reported to be continuously improved. In the future, in order to make this study draw more clinically instructive conclusions, it is still necessary to carry out high-quality clinical trials with multicenter, large samples and reasonable design methods and select internationally recognized outcome indicators.

## Figures and Tables

**Table 1:** The basic features of the included study (acupuncture as a monotherapy).

Author (year)	No. of participants (T/C)	Mean age or age range (T/C)		Treatment	Control	Outcome measures	Adverse events
Zhang (2021) [18]	39/41	47.95 ± 11.07	49.00 (41)	Acupuncture + sham medicine	Lornoxicam: 8 mg + sham acupuncture	Response rate VAS (10 min)	T: frequent urination (1); C: nausea (2); dizziness (1)
Kaouthar (2018) [13]	54/61	42 ± 14.8	41.8 ± 13.21	Acupuncture	Morphine: 0.1 mg/kg	Response rate and time of complete pain relief	T: needle blockage (1); itching/rash/bleeding at insertion point (2); C: dizziness (26); nausea and vomiting (13); drowsiness (1); rash (1); hypotension (1)
Xiao (2016) [26]	20/20	39.00 ± 12.31	39.6 ± 10.56	Acupuncture	Flurbiprofen Axetil: 50 mg	VAS (10, 30, 120 min) and time duration before pain remission	NR
Mehmet (2015) [12] II, I	41/40	42.39 (18–71)	46.3 (19–81)	Acupuncture	Acetaminophen: 1 g	VAS (10, 30, 60, 120 min)	T: none; C: allergic reaction (1); dizziness with vomiting (1)
Mehmet (2015) [12] II, III	41/40	42.39 (18–71)	37.98 (18–72)	Acupuncture	Diclofenac: 75 mg	VAS (10, 30, 60, 120 min)	T: none; C: rash (1); abdominal burning/pain (2)
Ju (2012) [24]	80/80	39 ± 11	39 ± 11	Acupuncture	Scopolamine: 20 mg	Response rate and time duration before pain remission	T: none; C: nausea (17); dizziness (33); fatigue (29); postural hypotension (12)
Yang (2011) [23]	27/27	—	—	Acupuncture	Fortanodyn: 100 mg	Response rate VAS (10, 30, 60, 120 min) and time duration before pain remission	T: none; C: not specified (6)
Xiang (2008) [22]	60/60	—	—	Acupuncture	Anisodamine: 10 mg + Tramadol: 100 mg	Response rate and time duration before pain remission	NR
Qiu (2006) [21]	30/30	26.7(23–56)	28.3(21–63)	Acupuncture	Tramadol: 100 mg + Atropine: 0.5 mg	Response rate	NR
Xu (2003) [20]	25/27	28.32 ± 6.56	27.26 ± 6.16	Acupuncture	Fortanodyn: 100 mg	Response rate, time duration before pain remission, and time of complete pain relief	NR
Li (1993) [19]	25/27	19–55	19–58	Acupuncture	Atropine: 0.5 mg	Response rate	NR
					Phenergan: 25 mg		
					(Fortanodyn: 100 mg)		

**Table 2:** The basic features of the included study (acupuncture as an adjuvant therapy).

Author (year)	No. of participants (T/C)	Mean age or age range (T/C)		Treatment	Control	Outcome measures	Adverse events
Sun (2021) [29]	30/30	38.93 ± 7.95	38.52 ± 8.23	Acupuncture + Phloroglucinol: 120 mg	Pethidine: 50 mg + Phloroglucinol: 120 mg	Response rate VAS (10, 30 min)	NR
Xiao (2020) [28]	38/38	37.42 ± 4.18	37.52 ± 4.31	Acupuncture + Anisodamine: 5–10 mg	Anisodamine: 5–10 mg	Response rate VAS (60 min)	NR
lu (2018) [27]	30/30	—	—	Acupuncture + Anisodamine: 10 mg	Tramadol: 100 mg + Anisodamine: 10 mg	Response rate	NR
Huang (2016) [25]	40/40	36.2	38.1	Acupuncture + Pethidine: 50 mg	Pethidine: 50 mg	Response rate	NR
Liu (2021) [30]	40/40	46.7 (13.11)	44.8 (14.6)	Acupuncture + Diclofenac: 50 mg	Sham acupuncture + Diclofenac: 50 mg	Response rate VAS (10, 30, 60 min)	T: none; C: none

**Table 3:** Details of needling (STRICTA).

Author (year)	Number of needles	Names or location of points	Depth of insertion	Responses sought	Needle stimulation	Needle retention time	Needle type
Zhang (2021) [18]	4	SP6, SP9	1cun, vertically inserted	Deqi	Manual	20 min	Aseptic acupuncture needle, specification 0.16 mm × 100 mm, Taixing Tianhe Medical Instrument Co., LTD. Producing Certificate of Jiangsu Province Food & Drug Administration: No. 2013–0073.
Sun (2021) [29]	—	GB25, BL23, BL40	GB25 (0.5–1.0 cun), BL23 (1–1.2 cun), BL40 (1.0–1.5 cun), vertically inserted	Deqi	Manual, stimulate per 10 min	30 min	Disposable acupuncture needles (Hwato)
Xiao (2020) [28]	—	GB25, BL23, SP6, PC6, pain sensitive	—	Deqi	Manual, may stimulate per 5 min	15–30 min	—
Kaouthar (2018) [13]	Left to the discretion of the physician, perhaps 2–16	BL21, BL22, BL23, BL24, BL26, BL45, BL46, BL47, BL48, BL49	1-2 cm, vertically inserted	Deqi	Manual	20 min	Sterile acupuncture needles were used (0.25 × 50 mm)
lu (2018) [27]	—	BL23, SP6, ST36	BL23(1.2–1.5cun), vertically inserted	Deqi	Manual, stimulate per 5 min	15–20 min	—
Xiao (2016) [26]	—	Qiu's point	—	Deqi	Manual, dragon—tiger fighting needling method will be performed on needles per 5 min	15 min	Sterile disposable acupuncture needles (0.3 × 40 mm; Hwato, Suzhou, China)
Huang (2016) [25]	—	GB25, BL23, SP6, PC6, pain sensitive point	—	Deqi	Manual, stimulate per 5 min	15–30 min	—
Mehmet (2015) [12]	—	BL21, BL22, BL23, BL24, BL45, BL46, BL47, BL48	—	Deqi	Manual	—	Sterile acupuncture needles (0.25 × 25 mm)
Ju (2012) [24]	—	ST36, PC6	—	Deqi	Manual, stimulate per 5 min	30 min	Stainless steel acupuncture needles (0.35 × 40 mm; 0.35 × 25 mm, hwato)
Yang (2011) [23]	—	KI3, BL60, KI2, BL63	KI3, BL60, 0.5cun				
1cun, vertically inserted	—	Manual	—	—			
Xiang (2008) [22]	—	BL23, BL40, ST36, Ashi point	—	—	Manual	—	—
Qiu (2006) [21]	—	ST36	—	Deqi	Manual	30 min	—
Xu (2003) [20]	—	BL23, BL40, ST36	—	Deqi	Manual	25–40 min	—
Li (1993) [19]	—	BL23, BL40, ST36	—	Deqi	Manual	25–40 min	—
Liu (2021) [30]	4	EX-UE7	Vertically inserted, 0.5 cun	Deqi	Manual	30 min	Sterile disposable stainless steel acupuncture needles (0.35 × 40 mm; Hwato, Suzhou, China)

## Data Availability

The data used to support the results of this study are available from the corresponding authors upon request.

## Supplementary Materials

Supplementary Materials The authors have completed the PRISMA reporting checklist (refer to Supplementary Appendix).
